# Cholangioscopy-guided laser lithotripsy alongside a plastic stent for common bile duct stones after total gastrectomy

**DOI:** 10.1055/a-2761-0266

**Published:** 2026-01-15

**Authors:** Ryo Soma, Haruo Miwa, Kazuki Endo, Ritsuko Oishi, Yuichi Suzuki, Hiromi Tsuchiya, Shin Maeda

**Affiliations:** 1 26437 Gastroenterological Center, Yokohama City University Medical Center, Yokohama, Japan; 2 Department of Gastroenterology, Yokohama City University Graduate School of Medicine, Yokohama, Japan

**Fig. 1:**
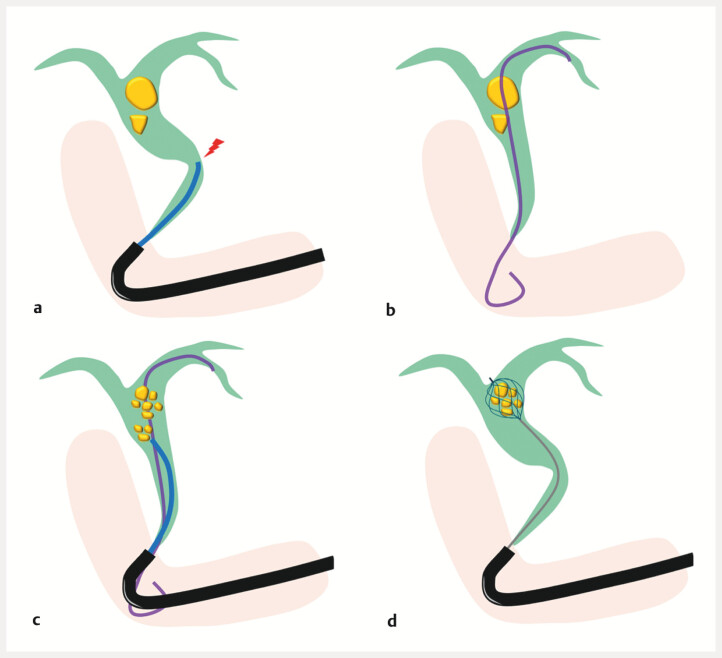
Schemas of peroral cholangioscopy (POCS)-guided lithotripsy performed alongside a plastic stent. **a** Maneuverability of the cholangioscope is limited when the common bile duct is highly angulated. **b** In such cases, anchoring a plastic stent in the intrahepatic bile duct can straighten the common bile duct. **c** Inserting the cholangioscope alongside this stent improves maneuverability during POCS-guided lithotripsy. **d** Stone extraction is facilitated following POCS-guided lithotripsy of the large stone.

Peroral cholangioscopy (POCS)-guided lithotripsy in patients with surgically altered anatomy is challenging 1 2 . Recently, a novel slim cholangioscope (9-Fr eyeMAX; Micro-Tech, Nanjing, China) has been developed that facilitates POCS-guided lithotripsy under balloon-enteroscopy assisted endoscopic retrograde cholangiopancreatography (BE-ERCP 3 4 ). However, the maneuverability of the cholangioscope is limited when the common bile duct is highly angulated. In such cases, inserting the cholangioscope alongside a plastic stent (PS) can improve maneuverability ( Fig. 1 ). In addition, the PS allows excess saline to flow out of the bile duct, thereby preventing cholangitis. Herein, we report a successful case of POCS-guided laser lithotripsy performed alongside a PS in a patient with common bile duct stones after total gastrectomy ( Video 1 ).

Cholangioscopy-guided laser lithotripsy alongside a plastic stent was performed for common bile duct stones in a patient after total gastrectomy.Video 1

**Fig. 2:**
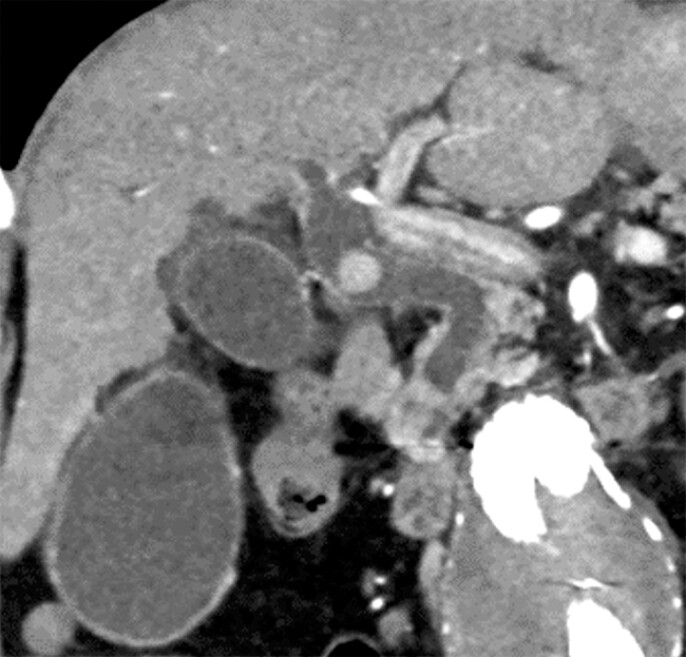
Computed tomographic image showing a 15-mm stone in a highly angulated common bile duct.

**Fig. 3:**
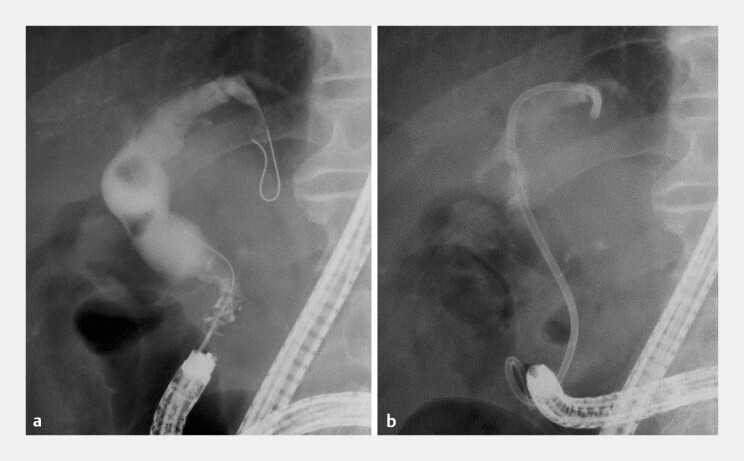
Fluoroscopic images of biliary stenting. **a** Biliary cannulation is performed using a rotatable papillotome, and the common bile duct is highly angulated. **b** A double-pigtail plastic stent (7-Fr, 12 cm) is placed with its proximal end anchored in the intrahepatic bile duct.

**Fig. 4:**
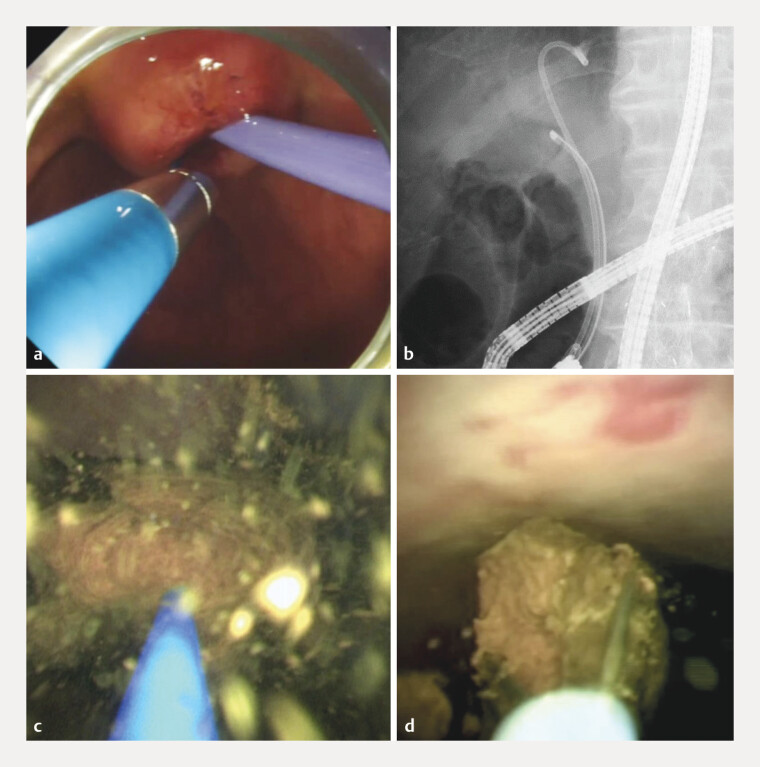
**a** The 9-Fr eyeMAX cholangioscope is inserted alongside the plastic stent. **b** The 9-Fr eyeMAX cholangioscope is advanced easily to the perihilar bile duct. **c** Laser lithotripsy is successfully performed using a holmium-YAG laser system (LithoEVO, Edap TMS). **d** A residual stone is removed using a micro-basket catheter under cholangioscopy guidance.

An 82-year-old man who had undergone total gastrectomy with Roux-en-Y reconstruction was referred to our hospital with cholangitis caused by large common bile duct stones ( Fig. 2 ). First, BE-ERCP was performed for biliary drainage. The bile duct was highly angulated, and a double pigtail PS (7-Fr, 12 cm REGULUS double pigtail; Japan Lifeline, Co., Ltd, Tokyo, Japan) was placed with its proximal end anchored in the intrahepatic bile duct ( Fig. 3 ). Six days later, POCS-guided lithotripsy was performed ( Fig. 4 ). Cholangiography demonstrated that the bile duct was straightened. Subsequently, the 9-Fr eyeMAX cholangioscope was inserted alongside the PS and advanced easily to the perihilar bile duct. Laser lithotripsy was successfully performed using a holmium-YAG laser system (LithoEVO; Edap TMS, Lyon, France). After lithotripsy, the stent was removed, and stone extraction was performed using a basket catheter. Because the bile duct remained angulated, the 9-Fr eyeMAX cholangioscope was reinserted with difficulty. A residual stone was removed using a micro-basket catheter under cholangioscopy guidance. The patient was discharged without complications.

To the best of our knowledge, this is the first report of POCS-guided lithotripsy performed alongside a PS, which improves the maneuverability of the cholangioscope.

Endoscopy_UCTN_Code_TTT_1AR_2AH
